# Towards the elimination of leprosy in Yunnan, China: A time-series analysis of surveillance data

**DOI:** 10.1371/journal.pntd.0009201

**Published:** 2021-03-16

**Authors:** Tie-Jun Shui, Heng Long, Li Xiong, Xiao-hong Zhang, Jun He, Xiaohua Chen

**Affiliations:** 1 Yunnan Center for Disease Control and Prevention, Yunnan, China; 2 Wenshan Institute of Dermatology, Wenshan Dermatology Hospital, The Alliance Hospital of the First Affiliated Hospital of Kunming Medical University, Yunnan, China; 3 Beijing Tropical Medicine Research Institute, Beijing Friendship Hospital, Capital Medical University, Beijing, China; 4 Beijing Key Laboratory for Research on Prevention and Treatment of Tropical Diseases, Capital Medical University, Beijing, China; Federal University of Ceará, Fortaleza, Brazil, BRAZIL

## Abstract

**Background:**

This study reviews the progress of leprosy elimination in Yunnan, China, over the past 30 years and identifies the challenges for the next stage of the program.

**Methodology/Principal findings:**

Data were collected from the Leprosy Management Information System in China (LEPMIS). The progress made in the elimination of leprosy between 1990 and 2019 was measured. We defined two time periods, time period 1 (1990–2003) and time period 2 (2004–2019), because multidrug therapy (MDT) was launched for the treatment of leprosy in 1990 and a special fund from the central government was established for leprosy in 2004. During the past 30 years, the number of newly detected leprosy patients in Yunnan has steadily declined. In total, 703 newly detected leprosy patients were reported in 1990, and 353 and 136 cases were reported at the end of 2003 and 2019, respectively. At the end of 1990, 90.7% (117/129) of counties in Yunnan Province were identified as leprosy-endemic counties (>1 case per 100,000 population). By the end of 2003 and 2019, 39.3% (46/117) and 85.5% (100/117) of the leprosy-endemic counties, respectively, had dropped below the elimination threshold. The main challenges are the remaining leprosy-endemic counties, the high rate of cases with a contact history, insufficient early detection, and leprosy cases resulting in physical disability.

**Conclusions/Significance:**

A multifaceted strategy for leprosy elimination in Yunnan Province has been successfully implemented, and remarkable progress has been made in the elimination of leprosy in this area. The priorities for leprosy elimination in the next stage are securing sustainable support and investment from the government, establishing an effective surveillance system, ensuring prompt early detection, providing treatment with MDT, preventing transmission of *M*. *leprae*, preventing disability, providing health education, and preventing recurrence of the epidemic situation of leprosy.

## Author summary

Compared to other provinces in China, Yunnan bears a significant burden of leprosy disease. Multidrug therapy (MDT) was launched for leprosy in 1990, and a special fund for leprosy was established in Yunnan Province by the central government in 2004. During the past 30 years, the number of newly detected leprosy patients has steadily declined, accompanied by an enormous decrease in the number of leprosy-endemic regions. Compared to the 1990–2003 period, the period after the establishment of the special fund from the central government (2004–2019) saw a decrease in the proportion of child cases and patients with grade 2 disability (G2D) and in the duration of delay in diagnosis among newly detected patients, while the proportion of patients with paucibacillary (PB) leprosy, the proportion belonging to minority groups, and the proportion of cases detected early increased significantly. The main challenges are the remaining leprosy-endemic counties, the high rate of cases with a contact history, insufficient early detection, and cases resulting in G2D. To eliminate leprosy in Yunnan Province, a multifaceted strategy should be implemented.

## Introduction

Leprosy, which is one of the oldest diseases known to humankind and is caused by *Mycobacterium leprae*, remains an important public health issue worldwide. According to the most recent data available from the World Health Organization (WHO), the reduction in the number of new cases has been gradual but consistent over the past 10 years. Globally, 202,185 new cases were detected in 2019 for a new case detection rate of 25.9 per million population, representing a global decrease of 6,506 cases. The registered prevalence of leprosy (point prevalence), i.e., the number of cases being treated at the end of 2019, was recorded as 177,175, with a corresponding prevalence rate of 22.7 per million population [1].

Historically, leprosy was endemic in China for more than 2,000 years, and the disease burden was high in the early days of the People’s Republic of China; this had a serious impact on people’s health and inhibited socioeconomic development [2]. After many years of intensive effort, leprosy has been effectively controlled in China through the implementation of effective strategies and measures, including sustainable support and investment from the government, the establishment of an effective surveillance system, management of leprosy patients, and treatment of leprosy, first with dapsone (DDS) monotherapy and then, after 1990, with multidrug treatment (MDT). The intensity of leprosy epidemics and the number of endemic areas have decreased significantly. In 2018, the number of leprosy cases nationwide was 521, a reduction of 54.4% compared with the 1,143 cases reported in 2011 [3].

Despite the declaration of the elimination of leprosy at the national level in 1998 (<1/100,000), leprosy remains prevalent in some of the difficult-to-access mountain regions of Southwest China [2,4]. To ensure progress toward the elimination of leprosy, a special fund was established by the Chinese government in 2004. In 2011, the Chinese government developed the National Leprosy Elimination Program (NLEP), which was officially endorsed that same year by the Ministry of Health. The general goal of the NLEP is to “control the leprosy epidemic effectively, eliminate the harm of leprosy, protect people’s health and promote the harmonious development of the economy and society”. According to the NLEP, the government of Yunnan Province developed the Leprosy Elimination Program in Yunnan Province (2011–2020) with the general goal of eliminating the harms of leprosy as quickly as possible and protecting the health of the people.

To understand the current status of the leprosy elimination effort in Yunnan Province and ensure the realization of the goal of further reducing the leprosy burden province-wide by 2020, in this paper, we review the achievements and experiences of the past 30 years and identify the challenges and priorities for future initiatives.

## Methods

### Ethical considerations

Ethical approval was not required because the analysis of LEPMIS data is a routine public health practice. Individual identifying information was not available and therefore not used.

### Profile of Yunnan

Yunnan Province is located in Southwest China, bordering Myanmar, Laos, and Vietnam. Covering approximately 394,000 square kilometers, Yunnan has a population of 47.71 million and is known for its high level of ethnic diversity. Yunnan consists of sixteen prefecture-level divisions (eight prefecture-level cities and eight autonomous prefectures), with 129 counties in total.

### Data collection

Data covering January 1^st^, 1990, to December 31^st^, 2019, were obtained from the Leprosy Management Information System in China (LEPMIS). The dataset comprises basic information on newly detected and relapsed leprosy cases, including patients’ basic demographic information (sex, date of birth, ethnicity, education, occupation, and geographic information) and clinical information (age at confirmed diagnosis, date of symptom onset, date of confirmed diagnosis, duration from the onset of symptoms to confirmed diagnosis, detection mode, grade of physical impairment, Ridley-Jopling classification, WHO operational classification). The study years were determined based on the launch of MDT in Yunnan (time period 1: 1990–2003) and the establishment of the special fund for leprosy by the central government (time period 2: 2004–2019). The new case detection rate (NCDR) was defined as the number of newly detected cases per year per 100,000 of the general population. The term “early detection” was used if the time between disease onset and diagnosis was within 2 years and the patient had Grade 0 or Grade 1 disability according to the WHO definition of leprosy disability [5].

### Data analysis and mapping

All data were entered into Microsoft Excel 2007 (Microsoft Corporation, Redmond, WA, USA), and descriptive analysis was performed. The data were subsequently analyzed using GraphPad Prism version 6 (GraphPad Software, La Jolla, California, USA) and OriginPro 2015 (OriginLab Corporation, Northampton, MA, USA). The chi-squared (and Fisher’s exact) test and the calculation of the odds ratio (OR) and 95% confidence interval (95% CI) were applied to identify differences in the demographic and clinical characteristics between time periods 1 and 2. Maps showing the geographical distribution of newly detected leprosy patients were generated using the inverse distance weighted interpolation method (IDW) in ArcGIS software version 10.1 (Environmental Systems Research Institute, Inc., Redlands, CA, USA).

## Results

### Demographic profile of leprosy, 1990–2019

A total of 11,052 newly detected cases of leprosy were reported in Yunnan from 1990 to 2019, with the NCDR per 100,000 ranging from 1.93 to 0.38. The NCDR decreased from 1.93 in 1990 to 0.91 in 1997, which was the first year that the goal of leprosy elimination was achieved in Yunnan. However, the NCDR increased again to 1.84 in 1998 and 1.19 in 1999. Since that time, the NCDR per 100,000 in Yunnan has remained under 1 and has continued to decline; it was most recently reported at 0.28 in 2019 (Table 1).

**Table 1:** Trends in Leprosy Elimination Indicators in Yunnan, China, from 1990 to 2019.

Year	New cases detected	Relapsed cases detected	NCDR per 100,000 population	MB proportion (%)		Children proportion (%)		Female proportion (%)		G2D proportion (%)		G2D rate per 100,000 population
Total	11052	514	0.89	7330	66.3%	452	4.1%	3346	30.1%	2356	21.3%	0.19
1990	703	44	1.93	463	65.9%	26	3.7%	195	27.7%	197	28.0%	0.54
1991	590	23	1.58	404	68.5%	22	3.7%	148	25.1%	147	24.9%	0.39
1992	621	30	1.65	413	66.5%	29	4.7%	170	27.4%	160	25.8%	0.42
1993	479	36	1.26	351	73.3%	17	3.5%	130	27.1%	124	25.9%	0.33
1994	479	25	1.25	336	70.1%	21	4.4%	136	28.4%	126	26.3%	0.33
1995	433	26	1.12	316	73.0%	16	3.7%	111	25.6%	120	27.7%	0.31
1996	494	26	1.27	336	68.0%	23	4.7%	150	30.4%	107	21.7%	0.28
1997	362	22	0.92	256	70.7%	22	6.1%	127	35.1%	83	22.9%	0.21
1998	735	21	1.85	409	55.6%	74	10.1%	221	30.1%	124	16.9%	0.31
1999	491	22	1.19	337	68.6%	22	4.5%	151	30.8%	89	18.1%	0.22
2000	382	15	0.94	268	70.2%	16	4.2%	113	29.6%	71	18.6%	0.17
2001	388	17	0.94	247	63.7%	17	4.4%	117	30.2%	75	19.3%	0.18
2002	358	18	0.86	232	64.8%	13	3.6%	101	28.2%	66	18.4%	0.16
2003	353	11	0.85	248	70.3%	13	3.7%	121	34.3%	61	17.3%	0.15
2004	337	22	0.80	217	64.4%	15	4.5%	104	30.9%	67	19.9%	0.16
2005	407	9	0.91	265	65.1%	11	2.7%	124	30.5%	70	17.2%	0.16
2006	366	8	0.82	224	61.2%	11	2.5%	117	32.0%	74	20.2%	0.16
2007	344	11	0.76	230	66.9%	4	1.2%	102	29.7%	66	19.2%	0.15
2008	393	16	0.86	232	59.0%	12	3.1%	114	29.0%	63	16.0%	0.14
2009	321	20	0.70	228	71.0%	8	2.5%	116	36.1%	62	19.3%	0.14
2010	228	4	0.50	135	59.2%	7	3.1%	72	31.6%	73	32.0%	0.16
2011	283	10	0.62	172	60.8%	7	2.5%	103	36.4%	83	29.3%	0.18
2012	230	16	0.50	147	63.9%	10	4.3%	72	31.3%	54	23.5%	0.12
2013	241	9	0.52	155	64.3%	6	2.5%	81	33.6%	43	17.8%	0.09
2014	208	10	0.44	136	65.4%	6	2.9%	68	32.7%	38	18.3%	0.08
2015	187	6	0.40	122	65.2%	10	5.3%	56	29.9%	30	16.0%	0.06
2016	170	11	0.36	109	64.1%	8	4.7%	56	32.9%	28	16.5%	0.06
2017	159	11	0.33	112	70.4%	1	0.6%	52	32.7%	20	12.6%	0.04
2018	174	9	0.36	129	74.1%	3	1.7%	67	38.5%	18	10.3%	0.04
2019	136	6	0.28	101	74.3%	2	1.5%	51	37.5%	17	12.5%	0.04

NCDR = New case detection rate, MB = Multibacillary, G2D = Grade 2 disability.

Among the new cases of leprosy detected, the percentage of multibacillary (MB) cases was relatively high in most of the years investigated, ranging from 59.0% in 2008 to 74.3% in 2019 (Table 1). The percentage of pediatric cases fluctuated between 0.6% in 2017 and 6.1% in 1997, except in 1998, when a peak of 10.1% was observed (Table 1). The percentage of female patients ranged from 25.1% in 1991 to 38.5% in 2018 (Table 1). Although the percentage of new cases with G2D ranged from 10.3% in 2018 to 32.0% in 2010 (Table 1), the G2D rate per 100,000 population consistently declined, from 0.54 in 1990 to 0.04 in 2019.

**Fig 1:**
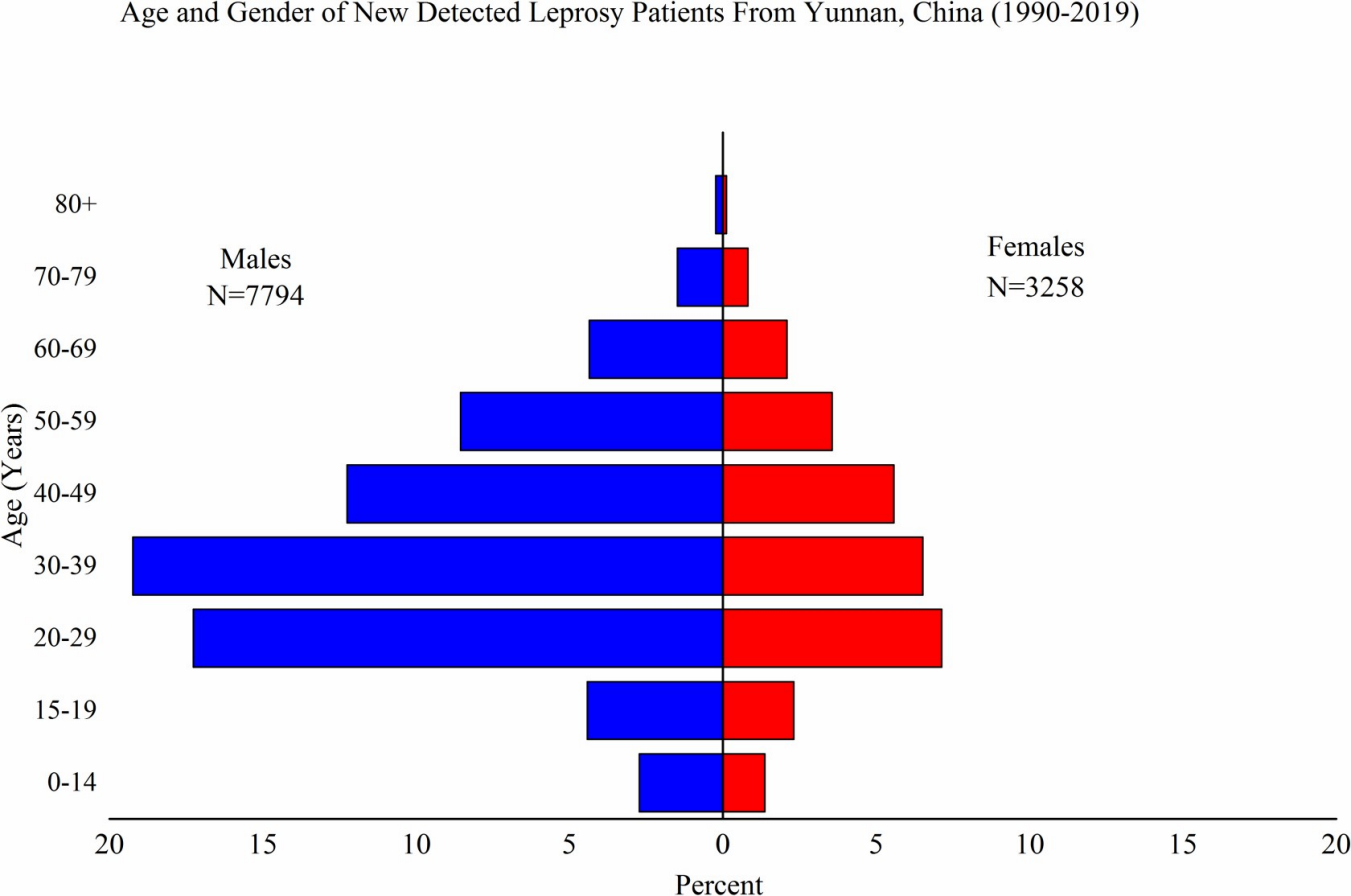
Demographic Profile of Newly Detected Leprosy Patients from Yunnan, China, 1990–2019. Age and sex of newly detected leprosy patients from Yunnan, China (1990–2019).

The age range for newly detected leprosy cases was broad (4–96 years), but cases were mainly concentrated in the 20–59 age group. The highest number of cases was reported for the 30–39 age group, which accounted for 25.6% (2,846/11,052) of all cases. The percentage of male patients was highest in the 30–39 age group (27.3%, 2,126/7,794), and the percentage of female patients was highest in the 20–29 age group (24.0%, 782/3,258) (Fig 1A). The proportion of males was significantly higher (P<0.01) than that of females in all age groups, with a male-to-female ratio of 2.39:1.

Thirty different ethnic groups were represented in this study. Han (47.4%, 5,242/11,052), Yi (13.6%, 1,501/11,052), Miao (9.9%, 1,096/11,052), and Zhuang (9.3%, 1,029/11,052) were the four groups most commonly represented among the newly detected cases.

Fifteen different occupations were registered. The largest number of newly detected leprosy cases was among farmers (91.8%; 10,146/11,052), followed by students (3.2%; 358/11,052) and factory laborers (1.3%, 148/11,052).

With regard to education level (2010–2019), 51.3% (829/1,616) of patients had a primary school education, 36.1% (584/1,616) had a junior high school education, 4.8% (77/1,616) had a senior high school education, 2.7% (43/1,616) had a college education or higher, and 2.7% (44/1,616) had no education.

### Epidemiological profile of leprosy, 1990–2019

**Fig 2:**
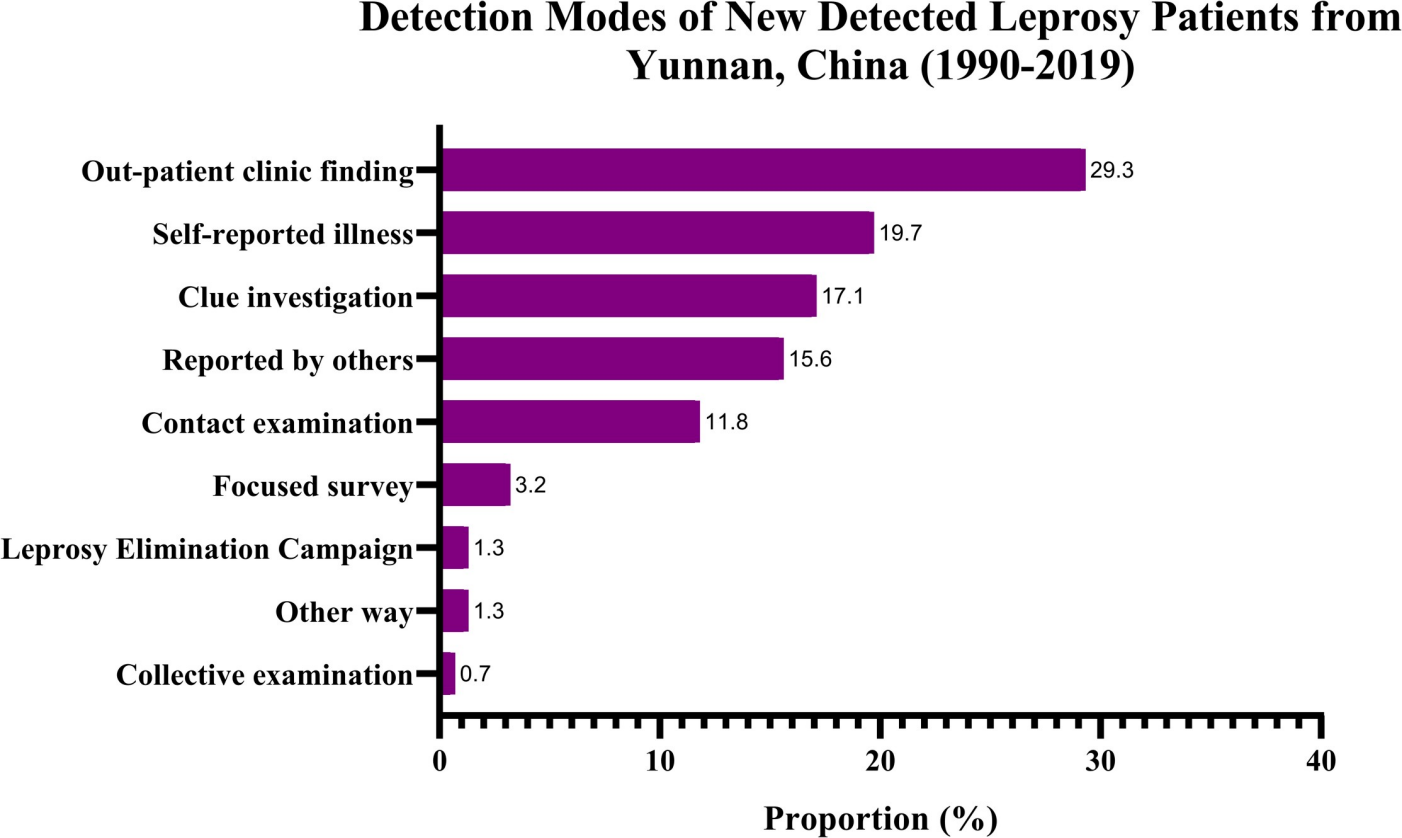
Detection Modes of Newly Detected Leprosy Patients from Yunnan, China, 1990–2019.

Among the 11,052 newly detected cases, the majority (7,031, 63.6%) were classified as MB leprosy and 4,021 (36.4%) were classified as PB leprosy (Table 2). In total, 7,533 (68.2%) had a history of close contact with a leprosy patient, 3,350 (30.3%) had contact with a leprosy patient within the family, and 4,183 (37.9%) had contact with a leprosy patient outside the family. The most common detection mode was outpatient clinic findings (2,983, 27.0%), followed by self-reported illness (2,279, 20.6%), clue investigation (1,963, 17.8%), reports by others (1,807, 16.4%), contact examination (1,272, 11.5%), focused surveys (in villages with leprosy cases) (343, 3.1%), other methods (157, 1.4%), leprosy elimination campaigns (LECs) (135, 1.2%), and group examinations (113, 1.0%) (Table 2 and Fig 2).

**Table 2:** Epidemiological Characteristics of Leprosy Cases Diagnosed in 1990–2019 in Yunnan, China.

Leprosy (n, %)		New detected			Relapsed		
		Total (1990–2019)	Period 1 (1990–2003)	Period 2 (2004–2019)	Total (1990–2019)	Period 1 (1990–2003)	Period 2 (2004–2019)
		11052 (100)	6868 (62.1)	4184 (37.9)	511 (100)	336 (100)	175 (100)
**Gender**	Male	7794 (68.1)	4928 (44.6)	2866 (25.9)	428 (83.8)	285 (84.8)	143 (81.7)
**Age**	< 15 years old	452 (4.1)	331 (3.0)	121 (1.1)	0 (0)	0 (0)	0 (0)
	≥ 15 years old	10603 (95.5)	6537 (59.1)	4066 (36.8)	511 (100)	336 (100)	175 (100)
**Classification**	MB	7031 (63.6)	4361 (39.5)	2670 (24.2)	442 (86.5)	290 (86.3)	152 (86.9)
	PB	4021 (36.4)	2507 (22.7)	1514 (13.7)	69 (13.5)	46 (13.7)	23 (13.1)
**Detection mode**	Spontaneous demand	2279 (20.6)	1536 (13.9)	743 (6.7)	84 (16.4)	40 (11.9)	44 (25.1)
	Dermatology	2983 (27.0)	1621 (14.7)	1362 (12.3)	58 (11.4)	34 (10.1)	24 (13.7)
	Report of illness by others	1807 (16.3)	1217 (11.0)	590 (5.3)	31 (6.1)	18 (5.4)	13 (7.4)
	Contact examination	1272 (11.5)	787 (7.1)	485 (4.4)	86 (16.8)	67 (19.9)	19 (10.9)
	Focus investigation	343 (3.1)	157 (1.4)	186 (1.7)	121 (23.68)	93 (27.7)	28 (16.0)
	Collective examination	130 (1.2)	100 (0.9)	30 (0.3)	1 (0.2)	0 (0)	1 (0.6)
	Clue investigation	1978 (17.9)	1252 (11.3)	726 (6.6)	74 (14.5)	54 (16.1)	20 (11.4)
	General survey	131 (1.2)	96 (0.9)	35 (0.3)	7 (1.2)	4 (0.1)	3 (0.2)
	Other ways	162 (1.5)	102 (0.9)	60 (0.5)	49 (9.6)	26 (7.7)	23 (13.1)
**Grade 2 disability**		2237 (20.2)	1441 (13.0)	796 (7.2)	190 (37.2)	123 (36.6)	67 (38.3)

MB: Multi-bacillary; PB: Pauci-bacillary.

### Implementation of leprosy elimination strategies and interventions

Since 1990, MDT has been used to treat leprosy in every county in Yunnan. In 2004, a special fund was established by the central government to combat leprosy in the region. The amount given from 2004 to 2019 was 150 million CNY (US$25 million). The strategies and interventions implemented to promote early detection of leprosy include a leprosy elimination program, symptom surveillance, contact examination, focused survey, training of medical staff, health education, and financial investments to improve transport and reconstruct the dermatology department and leprosy village.

### Comparison of new case detection statistics from the two time periods

The proportion of newly diagnosed patients compared to relapsed cases was nearly the same in both time periods, 95.3% and 96.0% (Table 3) at time period 1 and time period 2, respectively. Additionally, the statistics for MB leprosy, sex, and age did not change markedly between the two time periods. However, the number of newly diagnosed patients decreased from 6,868 in time period 1 to 4,184 in time period 2 (P<0.01) (Table 3). Interestingly, the percentage of newly detected patients belonging to an ethnic minority group increased from 51.4% (n = 3533) in time period 1 to 68.7% (n = 4181) in time period 2 (P <0.01). Additionally, early detection increased from 53.9% in time period 1 to 60.3% in time period 2 (P <0.01). The percentage of detected cases involving G2D decreased slightly from 22.6% (n = 1550) in time period 1 to 19.2% (n = 812) in time period 2 (P <0.01) (Table 3).

**Table 3:** Demographics and Prevalence of Newly Detected Leprosy Cases by Time Period in 1990–2003 and 2004–2019.

Variable	Time Period				OR	(95% CI)	P value
	1990–2003		2004–2019				
	N	(%)	N	(%)			
**Cases**							
**New**	6868	/	4184	/	1[Reference]		
**Population**	550,865,400	/	738,261,284	/	2.2	2.12 to 2.29	<0.01
**Cases**							
**New**	6868	95.3%	4233	96.0%	1[Reference]		
**Relapsed**	336	4.7%	178	4.0%	0.86	0.71 to 1.03	0.11
**Classification**							
**MB**	4616	67.2%	2752	65.0%	1[Reference]		
**PB**	2252	32.8%	1481	35.0%	1.1	1.02 to 1.20	0.02
**Gender**							
**Male**	4877	71.0%	2878	68.0%	1[Reference]		
**Female**	1991	29.0%	1355	32.0%	1.15	1.06 to 1.90	<0.01
**Age (years)**							
**≤14**	331	4.8%	121	2.9%	1[Reference]		
**>14**	6537	95.2%	4112	97.1%	1.72	1.39 to 2.13	<0.01
**Ethnicity**							
**Han**	3335	48.6%	1907	31.3%	1[Reference]		
**Minorities**	3533	51.4%	4181	68.7%	2.07	1.97 to 2.22	<0.01
**Early Detection**							
**Early Detection**	3700	53.9%	2522	60.3%	1[Reference]		
**Delay in Diagnosis**	3168	46.1%	1662	39.7%	0.77	0.71 to 1.25	<0.01
**Disability**							
**G2D**	1550	22.6%	812	19.2%	1[Reference]		
**Non G2D**	5318	77.4%	3421	80.8%	1.23	1.12 to 1.69	<0.01

*Differences among the study groups were evaluated using Fisher’s exact test for categorical data. Abbreviations: MB: Multi-bacillary; PB: Pauci-bacillary; OR: Odds ratio;CI: Confidence interval; G2D: Grade 2 disability.

### Decline in new case detection

With the introduction of MDT, the number of newly detected leprosy patients declined significantly. In 1990, a total of 703 newly detected leprosy patients were reported in Yunnan. This number decreased to 353 cases in 2003, a reduction of 50% in time period 1. After the special fund for leprosy was established by the central government in 2004, the number of newly detected leprosy patients decreased further to 136 cases in 2019, a decrease of 60% in time period 2 (Table 1).

### Continuous decline in the number of leprosy-endemic areas

**Fig 3:**
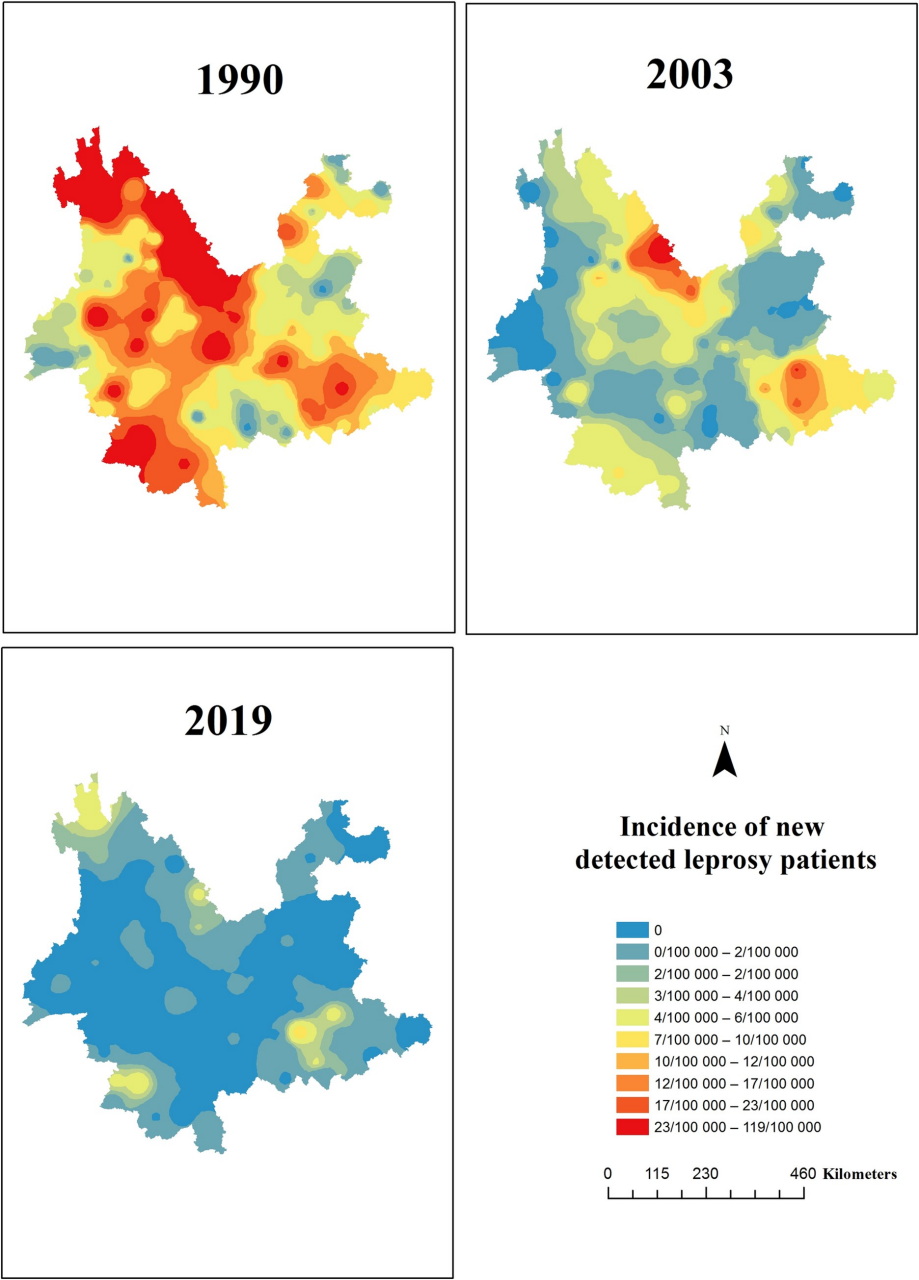
Changes in the Distribution of Newly Detected Leprosy Cases in Yunnan, China, 1990–2019.

Historically, newly detected leprosy patients have been reported in all 129 counties of the 16 prefecture-level divisions of Yunnan Province. In total, 127 counties reported newly detected leprosy cases, and 117 counties in Yunnan Province were identified as leprosy-endemic counties (>1 case per 100,000 population) when MDT was first launched in 1990. By the end of 2003, 39% (46/117) of the leprosy-endemic counties had achieved the leprosy elimination target. By the end of 2019, 85.5% (100/117) of the leprosy-endemic counties had achieved the leprosy elimination target (Fig 3). Currently, 17/117 (14.5%) of the counties remain leprosy-endemic regions.

## Discussion

In 2016, the WHO introduced the Global Leprosy Strategy 2016–2020, "Accelerating towards a leprosy-free world". The goal for 2020 was to further mitigate the global and local leprosy burden by reducing the number of new patients diagnosed with leprosy-related deformities (G2D) to fewer than one per million population [6]. This study is the first to retrospectively describe the substantial progress made in reducing leprosy and to analyze the challenges that lie ahead in Yunnan, a former hyperendemic region of leprosy in southwestern China. Compared to other provinces of China, Yunnan bears a significant leprosy burden. Multiple strategies have been used to make remarkable strides in reducing the leprosy burden over the past 30 years. In 2018, Yunnan met the basic standard for leprosy elimination at the provincial level for the first time (1 case per 100,000 population). Similar trends of consistent decline in leprosy case detection were also reported in the Republic of Korea [7], Taiwan [8], and Japan [9]. Despite these impressive achievements, challenges remain with respect to the control of leprosy in Yunnan.

When comparing the number of newly detected leprosy cases before and after the establishment of the special fund for leprosy by the central government, the observed reduction in the number of cases can be explained by socioeconomic development, improved access to health services, an effective surveillance system, better travel opportunities, and improved education for the population. Early detection has increased over the years and may have contributed to the observed slight reduction in the proportion of pediatric cases and patients with MB leprosy and G2D.

The progress made in terms of leprosy elimination has relied on multiple strategies. First, the Chinese government made concerted efforts in regard to leprosy prevention and treatment by promulgating and implementing a series of relevant policies, laws and strategies [10]. Second, success was supported by a number of scientific breakthroughs. Although dapsone demonstrated consistent activity against *M*. *leprae* by the early 1950s, dapsone resistance became widespread. Subsequently, MDT consisting of rifampin, dapsone, and clofazimine was recommended by the WHO in 1981 [11]. MDT was successfully introduced in Yunnan in 1983 [12]. In February 1983, Yunnan took the lead in a pilot study of MDT involving 47 leprosy patients in three leprosy villages in Mengla County [13]. In April 1986, based on the success of the trial of combined chemotherapy (also called MDT), the approach was rapidly extended to the whole province. By the end of 1989, all counties in the province had launched combined chemotherapy [12]. The combined chemotherapy program is ongoing. Third, a special fund for leprosy was established in the region by the central government in 2004, and a leprosy elimination program (2011–2020) was initiated in Yunnan to further eradicate the disease. Fourth, a surveillance system was developed and implemented in Yunnan to monitor and evaluate control and elimination activities. This system has played an important role in the efficient detection, treatment and management of leprosy cases and in the prompt elimination of infection sources. In addition, because health education is the principal component of most of the control and elimination programs [14], extensive training and health education was conducted for medical specialists, leprosy patients and the endemic population.

Challenges to achieving the goal of further reducing the local leprosy burden by 2020 are mainly related to helping the remaining 17 highly endemic counties reach the elimination target of less than 1 per 100,000 and preventing recurrence of the epidemic situation of leprosy by multiple means. The 17 highly endemic counties are heterogeneously distributed throughout the province. Understanding which geographical areas require intervention is fundamental for cost-effective disease control, and spatial analysis can indicate priority areas for local control programs [14,15]. Overcrowding, lower socioeconomic indicators, a lack of access to health services and precarious sanitation are underlying characteristics that impact the incidence of leprosy, indicating that poverty increases the likelihood of transmission of *M*. *leprae* [15].

Another challenge is that the majority (68.6%) of newly detected leprosy cases involved a history of close contact with a leprosy patient, typically outside the family. In terms of the mode of detection, only 12% of new cases were detected by contact examination. There is strong evidence that household and social contacts (at school, work, places of worship, etc.) and the neighbors of leprosy patients have a higher risk of leprosy than the general population [16–19]. Consequently, it has been suggested that contact surveys should not focus only on household contacts but should be extended to entire neighborhoods or villages to target a greater spectrum of social contact networks [15]. Contact tracing may not substantially influence the trend in new case detection [20,21] because leprosy patients are identifiable only when they have signs and symptoms of the disease, meaning that transmission might already have taken place during the asymptomatic stage [20]. The effect of contact tracing could be increased if asymptomatic contacts could be identified by a diagnostic test and subsequently treated or if postexposure chemoprophylaxis, such as single-dose rifampicin (SDR), which is currently recommended by the WHO, was provided to all contacts [22]. Mathematical modeling studies indicate that systematic contact tracing together with chemoprophylaxis for contacts can have a significant impact on the reduction in new cases in the long term [20]. Nevertheless, systematic contact tracing alone will benefit individuals with leprosy because they can be detected and treated earlier. This will interrupt the disease process in most cases, preventing severe nerve damage (G2D) [23]. Thus, it is necessary to focus on contact examination and chemoprophylaxis in the future. In addition, insufficient early detection of leprosy cases and G2D are challenges that must be addressed.

This study has some limitations. The LEPMIS was established by the National Center for Leprosy Control in 2010, and the data have since become more accurate and integrated. During the past several decades, the definitions for the WHO classifications of MB and PB have been continually adjusted. These classifications influence research and epidemiological studies. Due to the changes in the WHO classifications in the last several decades, it is difficult to compare work conducted 20 years ago with more recent work. Additionally, data across countries and even within a country are at risk of misclassification [24,25].

For the next stage in the elimination of leprosy in Yunnan, the following strategies should be emphasized. First, political commitment should be sustained at the national and local government levels, government leadership should be strengthened, sustainable funding should be provided, and routine and referral services should be strengthened within integrated health systems. Second, the quality of clinical services for early detection, diagnosis and treatment should be improved, especially by monitoring leprosy symptoms [26], as should the management of acute and chronic complications, including leprosy reactions and adverse drug reactions. Third, interventions for the prevention of disabilities/impairments and rehabilitation services should be launched as soon as possible. Fourth, scientific research should be conducted to promote new technologies for leprosy prevention, early detection, drug resistance monitoring, and relapse and case management. In addition, establishing and maintaining a surveillance system, utilizing LEPMIS, and strengthening the collection and utilization of epidemiological data can provide a scientific basis for formulating and perfecting prevention and control strategies for leprosy.

## Conclusion

The leprosy elimination activities conducted in Yunnan, China, over the past 30 years have been successful. The number of newly detected leprosy patients decreased greatly, and the number of highly endemic areas of leprosy decreased substantially, with only 17 counties remaining leprosy-endemic regions.
